# Enhancing Protocols for Concussion Management in Professional Soccer Events

**DOI:** 10.7759/cureus.64064

**Published:** 2024-07-08

**Authors:** Osvaldo Pangrazio, Francisco Forriol, Alex S Aguirre, Maria D Beletanga, Alcy R Torres

**Affiliations:** 1 Orthopedics, South American Football Confederation (CONMEBOL), Luque, PRY; 2 Family Medicine, South American Football Confederation (CONMEBOL), Luque, PRY; 3 Pediatrics, Boston University Chobania and Avedisian School of Medicine, Boston Medical Center, Boston, USA; 4 General Practice, Boston University Chobanian and Avedisian School of Medicine, Boston Medical Center, Boston, USA; 5 Pediatrics, Boston University School of Medicine, Boston, USA

**Keywords:** trauma, brain, concussion, incidence, soccer

## Abstract

Aim: Every year, there are an estimated 1.7 to 3.8 million sports-related traumatic brain injuries. A sports concussion results from an external force or a blow to the head or body, causing cranial encephalic trauma that can affect motor skills and brain function, producing varying symptoms related to an alteration in neurological functioning. Soccer poses a risk of concussions due to heading, where the player's head directly hits the ball to redirect or accelerate it. However, most concussions are caused by contact between players, such as head-to-head or head-to-elbow contact. This study analyzed the incidence of concussions or mild traumatic brain injuries in professional soccer during the “Copa America 2019” to understand the feasibility of soccer concussion protocols and propose evidence-based enhancements.

Methods: The data were previously collected by our first two authors, O. Pangrazio and F. Forriol, during the 46th edition of the “Copa America 2019,” where the South American Football Confederation implemented the Concussion Fast Recognition Protocol to detect traumatic brain injuries. The descriptive basic data will help to raise awareness and motivate further research in this field. We have analyzed and correlated it with global data to provide a comprehensive review and tangible evidence of the population size where soccer protocols are typically applied, thus calculating incidence rates to measure it mathematically.

Results: Our study reveals that the incidence rate of concussions during the “Copa America 2019” was 5.3 per 1,000 athlete exposures, with a total of three concussions occurring among 567 players. Despite the effectiveness of current protocols in detecting concussions rapidly and accurately, the relatively low incidence rate at this level of professional competition poses a challenge to validating these protocols. These results indicate that while the protocols in place are efficient, the testing and validation of new tools and approaches would be more beneficial at different levels of play where the incidence rates of concussions are higher. In environments with a greater frequency of concussions, the robustness and reliability of these protocols can be more thoroughly evaluated, ensuring they provide optimal protection and care for athletes.

Conclusion: The incidence of concussions is low in professional soccer tournaments. Protocols are necessary to protect players and educate sports professionals. However, their validation is difficult given the low incidence of concussions at this level of competition. Our proposed protocol helps unify a basic approach in the field and an advanced approach in any emergency department, providing better detection of concussions and improved outcomes for players. This protocol should be validated in populations with higher incidence rates to demonstrate its effectiveness.

## Introduction

Every year, there are an estimated 1.7 to 3.8 million sports-related traumatic brain injuries (TBIs) [1]. A sports concussion results from an external force or a blow to the head or body, producing a cranial encephalic trauma that can affect motor skills and brain function, and produce varying symptoms related to an alteration in neurological functioning. Typically, symptoms resolve between one- and two-week post-concussion, following a period of mental and physical rest, although some residual symptoms may remain for months [1,2]. Soccer poses a risk of concussions due to heading, where the player's head directly hits the ball in an effort to redirect or accelerate it; however, most concussions are caused by contact between players, such as head-to-head or head-to-elbow contact [3]. Out of all soccer injuries, 22% are concussions [3]. While in other sports and not professional leagues, it may be up to 21.5 per 1,000 athletic exposures [4]. In a study involving 222 soccer players of both sexes, completing 470 questionnaires, the median hits to the head during two weeks were 44 and 27 for men and women, respectively [5]. These numbers indicate that soccer poses a concussion threat to both sexes, especially in amateur leagues.

In a survey of the injury rate for children, soccer had the highest injury rate per athlete exposure (AE) among seven to 13-year-olds [6]. A further study evaluating concussions among university American football and soccer players found that 62.7% of soccer players experience symptoms of a concussion for at least one year after the episode [7]. However, at the professional level, the incidence of concussions is very low, ranging between 0.03 and 0.07 concussions per 1,000 player-hours in men and 2.6 concussions per 1,000 player-hours in women's professional soccer [8,9].

Concussion management in professional soccer has become a pressing issue in recent years due to the growing awareness of the long-term consequences of head injuries. Despite advancements in sports medicine, the protocols for identifying and managing concussions in soccer remain insufficient. Our expert opinion explores the existing protocols, evaluates their effectiveness, and proposes enhancements to ensure the safety and well-being of athletes in professional soccer events. This study analyzed the incidence of concussions or mild TBIs in professional soccer during the “Copa America 2019” to understand the feasibility of soccer concussion protocols and propose evidence-based enhancements.

## Materials and methods

The data were previously collected by our first two authors, O. Pangrazio and F. Forriol [10], during the 46th edition of the “Copa America 2019” in Brazil, a continental event organized by the South American Professional Soccer Association (CONMEBOL), where the Concussion Fast Recognition Protocol (CFRP) was used to detect TBIs. Prior to the tournament, CONMEBOL prepared a file that was given to each team at their technical meetings to indicate how to use CFRP in the event of a concussion, but also mandatory reports were filled by the referee and the medical staff of each team. All the locker rooms had a sign with the protocol in three languages: Spanish, Portuguese, and English. Furthermore, every team filled out and delivered the CFRP after each match. Every injury was reviewed on-screen by replaying the corresponding minute of injury from the recorded gameplay.

The CFRP was to be used in case any players suffered or were suspected of having a concussion. If a concussion was suspected, the team’s health professional, with concussion training, assessed and evaluated the player. The injured player was removed from the game when necessary, and a report describing all the incident details was created, even if there was no concussion.

The data were collected from publicly available transmissions retrospectively. The descriptive basic data will help to raise awareness and motivate further research in this field. We have analyzed and correlated it with global data to provide a comprehensive review and tangible evidence of the population size where soccer protocols are typically applied, thus calculating incidence rates (IRs) to measure it mathematically.

Our study examined concussion IRs. Soccer players (n = 567) from 12 teams; 10 from CONMEBOL and two from the Asian Football Confederation (AFC) were followed. IRs for concussion per 1,000 AEs were calculated across the tournament.

## Results

The “Copa America 2019” was hosted in Brazil, featuring 26 matches and approximately 567 professional footballers who played for over 2,340 minutes. The tournament included 14 national teams over three weeks. During these matches, 40 injuries were observed and recorded. Among these, two injuries were due to infectious diseases, resulting in the isolation of two players - one with measles and the other with chickenpox. The remaining 38 were traumatic injuries.

**Figure 1:**
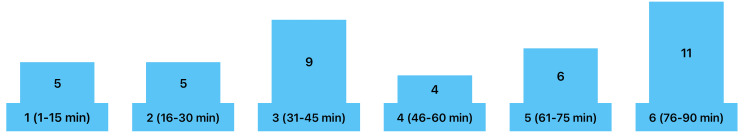
Injuries sorted by minute occurred Image Credits: Alcy R. Torres, MD

The median player age was 29 years old, with an age range of 21 to 35 and a standard deviation of four years. Of the injured players, 12 were defenders, 12 were forwards, 12 were midfielders, and two were goalkeepers. Most of the injuries occurred in the last quarter of the second half (minutes 76-90), as illustrated in Figure 1. 

The distribution of injuries highlights several critical factors in understanding player safety and injury prevention. Defenders and midfielders, who are typically involved in frequent physical confrontations and high-intensity actions, represented a significant portion of the injured players. The high incidence of injuries occurring towards the end of matches suggests fatigue and decreased vigilance as contributing factors.

Moreover, the data underscores the importance of robust medical protocols and emergency response plans during such high-stakes tournaments. It also emphasizes the need for ongoing research and the development of enhanced protective measures to mitigate the risk of both traumatic and infectious incidents.

By focusing on the timing and nature of these injuries, future protocols can be better tailored to address specific vulnerabilities, such as providing additional medical staff during the critical final minutes of play and implementing strategies to manage player fatigue. Additionally, the isolation cases due to infectious diseases highlight the necessity for stringent health monitoring and vaccination policies to prevent such occurrences. There were three concussions as detailed.

The first concussion occurred due to a collision between two players from the same team. One player was kneed in the head as the other fell to the ground. The injured player remained on the ground while the match continued until the referees were notified, prompting a pause in the game. The medical team observed the player experiencing seizures, disorientation, and an inability to answer standard concussion questions. This player was promptly taken to the hospital and kept under observation for 24 hours. Remarkably, he returned to play in the next game just four days later, highlighting the rapid recovery but also raising concerns about the adequacy of the rest period. 

The second concussion occurred when a player was struck by an opponent. The injured player initially lay on the field but eventually got up and continued to play. The team doctor later noticed the player exhibiting unusual neurological signs, such as moving in an atypical pattern. Upon further evaluation, the player displayed excessive aggressiveness in his responses and an inability to answer questions accurately. Despite the player's insistence on continuing to play, he was removed from the field and transferred to the hospital for observation. He did not participate in any further matches but was asymptomatic during the seven-day post-concussion evaluation. This case underscores the importance of medical intervention and the potential risks of continuing to play after a head injury.

The third concussion resulted from a head-to-head collision. Initially, the injured player did not appear to be affected and continued playing without showing immediate signs of distress. However, at the end of the game, the player appeared disoriented and was subsequently evaluated by the medical team. Despite the disorientation, the player remained asymptomatic. This incident highlights the challenges of detecting concussions immediately and the necessity for post-game evaluations. All three concussions were assessed using the CFRP, as outlined in the methodology section. These three concussions occurred over 2,340 minutes of play, resulting in an IR of 5.3 per 1,000 AEs. The concussions resolved within three days, with no residual symptoms.

## Discussion

Our data

The “Copa America 2019” had a low incidence rate of concussions, only accounting for three out of the 567 players participating. While this number is low, players could still have further complications related to post-concussion syndrome. The purpose of the CFRP is to aid in the recognition and management of professional soccer players. However, its validation may not be perfectly understood in a population with a low incidence of concussions.

Current protocols

The current protocols for concussion management in professional soccer typically involve a combination of on-field assessment, sideline evaluation, and off-field medical examination. When a player sustains a head injury, medical staff assess them on the field for visible signs of concussion, such as loss of consciousness or disorientation. However, relying solely on observable symptoms may not accurately diagnose concussions, as some symptoms may be subtle or delayed.

Following an on-field assessment, players suspected of concussion are often substituted and undergo further evaluation on the sideline. This evaluation typically involves cognitive and balance assessments, such as the SCAT6 (Sport Concussion Assessment Tool, Concussion in Sport Group, North America) [11]. However, the reliability of these tests in accurately diagnosing concussions remains a subject of debate.

If a concussion is suspected, players are typically removed from the game and undergo further medical evaluation off the field. This evaluation may include a complete neurological examination and sometimes neuropsychological testing to assess the severity of the injury and guide its management. However, access to advanced medical facilities and expertise may vary depending on the league and location of the event.

Proposed enhancements

While athletes are unable to completely avoid risky actions, especially within a contact sport, the future concern for athletes should be the prevention and urgent recognition of concussions. Within a team, all players and support staff should be educated to recognize the signs and symptoms of a concussion in order to refer the player to a medical professional for further examination. Even though soccer allows for a limited ability to take a prolonged break in order to do a detailed evaluation of a player, the establishment of video recording and artificial intelligence can be a helpful tool to begin a concussion evaluation or to diagnose the TBI [12].

To address these limitations, several enhancements to concussion protocols in professional soccer events are recommended. Firstly, there should be mandatory education and training for players, coaches, referees, and medical staff on recognizing the signs and symptoms of concussion. This can help promote a culture of safety and encourage players to report head injuries without fear of repercussions.

Secondly, objective measures, such as sideline concussion assessment tools and additional testing, should be integrated into the evaluation process to supplement subjective assessments. This can improve the accuracy of diagnosis and facilitate more consistent management across different events and leagues.

Additionally, independent medical professionals with expertise in concussion management should be involved in the evaluation and decision-making process to ensure impartiality and prioritize player welfare over competitive considerations. Clear guidelines should be established for the return-to-play process, with emphasis on gradual progression and individualized care based on the severity of the injury.

**Figure 2:**
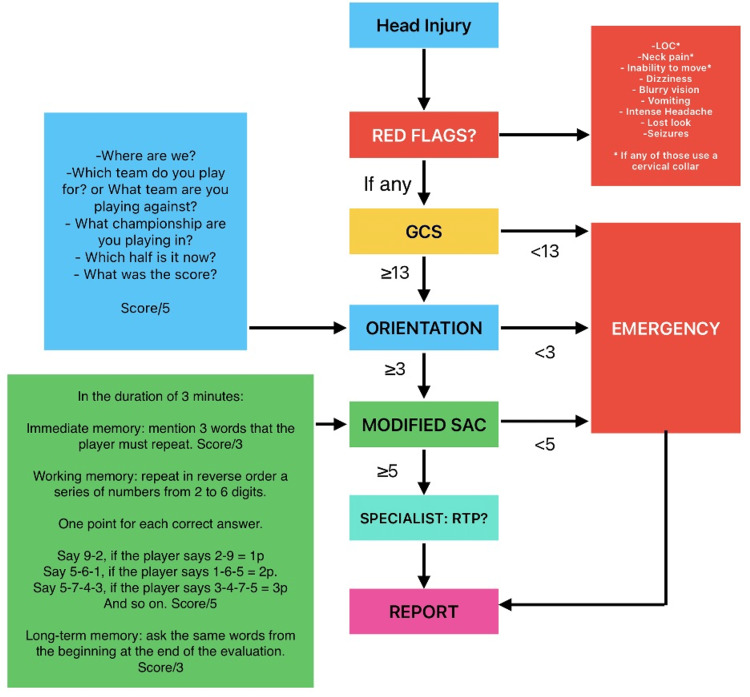
Proposed algorithm to improve concussion management in soccer events. GCS: Glasgow Coma Scale, SAC: standardized assessment of concussion, RTP: return to play. Image Credits: Alcy R. Torres, MD

Based on this data, we propose a new algorithm (Figure 2) that is based on the evaluation tools that are used in the emergency department to assess the severity of the concussion. This proposed algorithm uses some elements of the Glasgow Coma Scale (GCS), five orientation questions based on the SCAT6, and a modified standardized assessment of concussion (SAC) to direct the professional soccer player into observation or the emergency room [13]. Given that these newly proposed algorithms use previously validated tools, the feasibility and accuracy of detecting even minor concussions should improve. This algorithm considers the risky behaviors of professional soccer players of following unsafe rules to keep playing against medical advice by diminishing their feelings or symptoms [14,15]. Nevertheless, this proposed protocol should be validated for different ages, languages, types of games, and settings, such as training and games during competitions as mentioned.

We propose an algorithm that initially screens for red flags to use a cervical collar and to start further examination after a head injury. If any red flag is present, then a GCS should be applied to the player. If GCS is lower than 13, the patient should be directed to the emergency room for further examination, because it objectively shows the presence of a mild TBI [16]. If GCS is equal to or higher than 13, orientation questions should be applied to the player. If the orientation questions score is lower than 3, the patient should be directed to the emergency department (ED). If the player's orientation questions score is equal to or higher than 3, a modified SAC should be applied to the patient. If the patient's score on the modified SAC questions is less than five, the patient should be directed to the ED. If the modified SAC score is equal to or higher than five, the patient should be referred to a specialist to consider their ability to return to play. In any case, a report with all the data and the scores should be created and sent to the regulatory institution.

While current protocols aim to prioritize player safety, several restraints hinder their effectiveness. First, the reliance on subjective assessments of symptoms may lead to underreporting of concussions by players who fear being sidelined or stigmatized. Moreover, the lack of standardized diagnostic criteria and variability in medical expertise among team staff can result in inconsistent management practices. Furthermore, the pressure to win and the financial stakes involved in professional soccer may influence decision-making regarding players' return to play. In some cases, there may be a temptation to rush players back onto the field without adequate recovery time, increasing the risk of long-term complications.

Our study has some limitations, such as the lack of empirical data proving that our proposed algorithm would be effective in both amateur and professional soccer settings. However, by leveraging existing, proven medical algorithms and data, we can reasonably infer that our algorithm will be feasible and user-friendly in the field. Additionally, while our study primarily focused on a high-level professional tournament with a relatively low incidence of concussions, further research and testing in diverse settings with varying levels of play are necessary to validate and refine our approach. By doing so, we aim to enhance the generalizability and effectiveness of our concussion management protocols, ensuring they provide comprehensive protection and care for athletes at all levels of competition.

## Conclusions

The low IR of concussions in a highly competitive professional soccer event underscores the effectiveness of current protocols in detecting and managing concussions, enabling rapid assessment and appropriate medical intervention. However, the relatively low frequency of concussions at this level poses challenges in validating and refining these protocols, necessitating testing in environments with higher concussion rates to ensure their robustness. While existing protocols are efficient, there is a continuous need for enhancement to provide more comprehensive protection and better management strategies, especially during critical moments following an injury and in post-game evaluations. The incidents where players continued to play despite showing symptoms highlight the importance of strictly enforcing medical advice and protocols to prioritize players' long-term health and safety. Additionally, the study emphasizes the need for ongoing education for players, coaches, and medical staff about the risks and signs of concussions, promoting better on-field decision-making and adherence to safety protocols.

## References

[1] Conder RL, Conder AA (2015). Sports-related concussions. N C Med J.

[2] Moser RS, Glatts C, Schatz P (2012). Efficacy of immediate and delayed cognitive and physical rest for treatment of sports-related concussion. J Pediatr.

[3] Maher ME, Hutchison M, Cusimano M, Comper P, Schweizer TA (2014). Concussions and heading in soccer: a review of the evidence of incidence, mechanisms, biomarkers and neurocognitive outcomes. Brain Inj.

[4] Clay MB, Glover KL, Lowe DT (2013). Epidemiology of concussion in sport: a literature review. J Chiropr Med.

[5] Stewart WF, Kim N, Ifrah CS (2017). Symptoms from repeated intentional and unintentional head impact in soccer players. Neurology.

[6] Radelet MA, Lephart SM, Rubinstein EN, Myers JB (2002). Survey of the injury rate for children in community sports. Pediatrics.

[7] Delaney JS, Lacroix VJ, Leclerc S, Johnston KM (2002). Concussions among university football and soccer players. Clin J Sport Med.

[8] Dvorak J, McCrory P, Kirkendall DT (2007). Head injuries in the female football player: incidence, mechanisms, risk factors and management. Br J Sports Med.

[9] Waldén M, Hägglund M, Orchard J, Kristenson K, Ekstrand J (2013). Regional differences in injury incidence in European professional football. Scand J Med Sci Sports.

[10] Pangrazio O, Pagura J, Forriol F (2019). Analysis of the concussion fast recognition Protocol (CONMEBOL), in Copa America 2019, for the detection, evaluation and treatment of concussion. SLOAT.

[11] Echemendia RJ, Meeuwisse W, McCrory P (2017). The Sport Concussion Assessment Tool 5th Edition (SCAT5): Background and rationale. Br J Sports Med.

[12] Patricios JS, Ardern CL, Hislop MD (2018). Implementation of the 2017 Berlin Concussion in Sport Group Consensus Statement in contact and collision sports: a joint position statement from 11 national and international sports organisations. Br J Sports Med.

[13] McCrea M, Kelly JP, Kluge J, Ackley B, Randolph C (1997). Standardized assessment of concussion in football players. Neurology.

[14] Chen Y, Buggy C, Kelly S (2019). Winning at all costs: a review of risk-taking behaviour and sporting injury from an occupational safety and health perspective. Sports Med Open.

[15] Williams JM, Langdon JL, McMillan JL, Buckley TA (2016). English professional football players concussion knowledge and attitude. J Sport Health Sci.

[16] Mehta R, Chinthapalli K (2019). Glasgow coma scale explained. BMJ.

